# Input clustering and the microscale structure of local circuits

**DOI:** 10.3389/fncir.2014.00112

**Published:** 2014-09-12

**Authors:** William M. DeBello, Thomas J. McBride, Grant S. Nichols, Katy E. Pannoni, Daniel Sanculi, Douglas J. Totten

**Affiliations:** ^1^Center for Neuroscience, Department of Neurobiology, Physiology and Behavior, University of California-DavisDavis, CA, USA; ^2^PLOS MedicineSan Francisco, CA, USA

**Keywords:** synapse clustering, synaptic clustering, clustered plasticity, connectome, dendritic integration

## Abstract

The recent development of powerful tools for high-throughput mapping of synaptic networks promises major advances in understanding brain function. One open question is how circuits integrate and store information. Competing models based on random vs. structured connectivity make distinct predictions regarding the dendritic addressing of synaptic inputs. In this article we review recent experimental tests of one of these models, the input clustering hypothesis. Across circuits, brain regions and species, there is growing evidence of a link between synaptic co-activation and dendritic location, although this finding is not universal. The functional implications of input clustering and future challenges are discussed.

## The era of structural and functional connectomics

Since the discovery by Santiago Ramón y Cajal that the brain is a network, a central goal has been to map its wiring diagram. This remains a grand challenge. The human brain, for example, contains an estimated 100 trillion chemical synapses interconnecting ~80 billion neurons entangled within a volume of ~12 quadrillion cubic microns. Traditional methods that provide the nanometer scale resolution needed to reliably identify individual synapses have lacked the throughput capacity to reconstruct even a small portion of the connection matrix. Until this gap is bridged, a number of crucial issues relating to both normal and diseased brain states will remain unresolved (Crick and Jones, 1993).

Major steps towards the realization of Cajal’s vision are within sight. Technological advances over the past decade have brought the promise of mapping virtually every synaptic connection within local circuits close to reality (Briggman and Denk, 2006; Smith, 2007; Helmstaedter et al., 2008; Lichtman and Sanes, 2008; Lehrer, 2009; Kleinfeld et al., 2011; Briggman and Bock, 2012; Marc et al., 2012; Morgan and Lichtman, 2013). This is has been termed microscale connectomics (Sporns et al., 2005). The microscale distinction is important as related efforts are underway to create brain-wide maps of the axonal projections emanating from sub-nuclei, the mesocale connectome or projectome (Kasthuri and Lichtman, 2007; Bohland et al., 2009) and to chart larger inter-areal bundles visualized using MRI-based diffusion tensor tractography (Sporns, 2013), the macroscale connectome. The latter is the basis of the Human Connectome Project, launched in 2010, which promises insights into individual variability with potential to identify gross anatomical disturbances underlying a range of neurological disease (Behrens and Sporns, 2012). In comparison with microscale methods and microelectrode recording, however, the spatial and temporal resolution of macroscale connectomics is 6–7 orders of magnitude less than what is required to reliably identify individual synapses.

Herein the term connectome is used to denote the microscale, on the scale of nanometers and microseconds. Techniques for structural connectomics have matured over the last decade. Transmission electron microscopes (TEM) equipped with automated stage controllers and high-throughput detectors can image within weeks libraries of ultrathin sections that encompass entire local circuits (fixed tissue blocks < ~1 mm^3^). The contrast and resolution, ~2 nm lateral, are excellent, the historic gold standard for identifying synapses based on the presence of presynaptic vesicles, an intercellular cleft, and an electron-opaque postsynaptic density, or PSD (DeRobertis and Bennett, 1955; Palay and Palade, 1955; reviewed in Harris and Weinberg, 2012). Images can be registered in 3D using morphing algorithms (e.g., Anderson et al., 2009) to produce a volume for analysis. Serial block-face scanning electron microscopy (SBFSEM; Denk and Horstmann, 2004) uses back-scattered electrons to image the surface of a block, then shaves and discards the top ~30 nm to reveal a new surface. The images are in natural alignment and gaps are minimal. Focused ion beam scanning electron microscopy (FIBSEM), a technique borrowed from the semiconductor industry, uses ions instead of electrons to image and ablate the block surface (Knott et al., 2008, 2011; Merchán-Pérez et al., 2009), producing outstanding contrast and resolution. In addition to mapping synapses, all of these techniques permit reconstruction of complete neuronal morphologies and tracking of axons throughout the local volume; in some cases much further (Mikula et al., 2012).

Connectomics methods based on light microscopy (LM) are well suited for the mesoscopic level (Osten and Margrie, 2013), but can also reveal microscale connectivity through the visualization of intrinsic or genetically encoded synaptic markers. Array Tomography (AT; Micheva and Smith, 2007; Micheva et al., 2010) uses serial application of antibodies directed against endogenous proteins known to localize to vesicles or the PSD. Validated by correlative EM studies, synapse identification can be >80% reliable, which is extremely useful in the context of high-throughput capacity (Rah et al., 2013). CLARITY renders the entire brain optically transparent by fixing proteins to a hydrogel scaffold and then removing lipids. Images from deep in the tissue can be obtained without the need for physical sectioning, and proteins can be detected *in situ* using fluorescent probes (Chung and Deisseroth, 2013; Chung et al., 2013). Trans-synaptic tracing with neurotropic viruses is tightly restricted to synaptically connected neuronal ensembles (Callaway, 2008). GFP Reconstitution Across Synaptic Partners (GRASP; Feinberg et al., 2008) targets genetically encoded fragments of green fluorescent protein to pre- and post-synaptic membranes, effectively marking only sites of synaptic contact. In Brainbow mice, individual neurons express just one of ~100 different colors which substantially facilitates circuit analysis (Livet et al., 2007). These diverse approaches for introducing cell- and synapse- specific fluorescent labels may also benefit from super-resolution imaging methods (Hell, 2003, 2007; Rust et al., 2006) that circumvent the diffraction limit of light-based optics and provide enhanced resolution down to ~20 nm laterally.

Techniques for functional connectomics—the real-time activity history of every neuron/synapse in the volume—are lagging in terms of coverage density but include promising advances in optical recording using calcium or voltage-sensitive dyes, increasingly higher density electrode arrays with possibilities for nanoscale miniaturization, powerful optogenetic methods to directly probe circuit function (Boyden et al., 2005; Deisseroth, 2011), and novel strategies in earlier stages of technological development (reviewed in Alivisatos et al., 2012, 2013a,b). Further development of all these methods will likely get a major boost from the BRAIN Initiative (Brain Research through Advancing Innovative Neurotechnologies) funded by the U.S. National Institutes of Health. Collectively, existing and in-progress tools for structural and functional connectome analysis appear poised to produce a mountain of data in the near future.

This begs the question … what is the question?

Dense reconstruction—every synapse, every cell, every wire—is for now limited to small volumes <1 mm^3^, thus, a current challenge is to select circuits that are both physically compact and functionally sophisticated so that structure-function relationships can be tested. One recent success is identification of a candidate visual motion detection circuit in the Drosophila optic medulla (Takemura et al., 2013). In combination with new methods for recording from the unusually small neurons implicated (Maisak et al., 2013), a deeper understanding of how the fly detects visual motion appears imminent. Such an achievement would build on the legacy of connectomes mapped to date including the entire nervous system of the worm *C. elegans* (White et al., 1986; Jarrell et al., 2012) and canonical circuit motifs in the mouse neuromuscular junction (Lu et al., 2009b; Tapia et al., 2012), rat hippocampus (Mishchenko et al., 2010), rabbit retina (Anderson et al., 2011; Marc et al., 2013), mouse retina (Briggman et al., 2011; Helmstaedter et al., 2013), and mouse primary visual cortex (Bock et al., 2011). None of this could have been possible without parallel advances in annotation platforms and development of semi-automated segmentation pipelines with human error-checking (Fiala, 2005; Mishchenko, 2008; Lu et al., 2009a; Anderson et al., 2010; Chklovskii et al., 2010; Jain et al., 2010; Jeong et al., 2010a,b; Jurrus et al., 2010; Tasdizen et al., 2010; Turaga et al., 2010; Helmstaedter et al., 2011; Roberts et al., 2011; Beyer et al., 2013; Hu et al., 2013; Xu et al., 2013) and one nearly fully automated pipeline for synapse identification (Kreshuk et al., 2011). Still, a fast digital solution to the challenge of dense circuit reconstruction has proven elusive. Efforts towards this goal continue in parallel with online projects to distribute the annotation tasks to larger numbers of people by leveraging the attraction of video gaming, as in the citizen science websites Eyewire, WiredDifferently and SLASH (Scalable system for Large data Analysis and Segmentation utilizing a Hybrid approach).

To date, microscale connectomics has taken promising steps towards generating new understanding of circuit-specific computations. As technologies advance, a new challenge will be to elucidate general principles that operate across circuits, in particular, the capacity of biological networks to integrate and store information.

## Local connectivity in neural circuits: random or structured?

Circuits are packed with diverse cell types whose axons and dendrites intertwine in tight quarters. Understanding the degree to which pre- and post-synaptic partners exhibit specificity for one another—at the level of cell type, dendritic domain and dendritic address—has been a longstanding goal (Ramón y Cajal, 1954; Szentágothai, 1978; White, 2007). Analysis of projections from the lateral geniculate nucleus to visual cortex in rats led Peters and Feldman to postulate that the number of synapses made between two neurons is proportional to the geometric overlap between axon and dendrite (Peters and Feldman, 1976). This was termed Peters’ rule (Braitenberg and Schuz, 1991) and was extrapolated to a general principle of brain organization. In comparing predictions from geometric overlap of cortical pyramidal and stellate cells with actual synapse counts based on electron microscopy, Braitenberg articulated the concept of random connectivity:

“This play with probabilities is legitimate only if synapses between cortical neurons are made entirely by chance, depending only on the accident of some axon of one neuron coming into the immediate vicinity of some dendrite of another”. V. Braitenberg et al., *Cortex: Statistics and Geometry of Neuronal Connectivity, 1998*.

Peters’ rule has a practical implication. If precise connectivity can be inferred from geometric overlap of cells reconstructed in separate tissue blocks using standard approaches, then dense microscale reconstruction might be unnecessary (da Costa and Martin, 2013). In this view, efforts should instead focus on developing a complete catalog of cell types, statistics that capture fine details of morphometric variations, and computational strategies to properly register thousands of cells in 3D and ultimately calculate the synaptic network. Several projects have leveraged this strategy (Binzegger et al., 2004; Amirikian, 2005; Lang et al., 2011; Oberlaender et al., 2012; Ramaswamy et al., 2012). The degree to which such inferred networks correspond to anatomical ground truth, or how faithfully simulations based on their structure will robustly reproduce circuit function, remain important questions (Ascoli, 2012).

**Figure 1:**
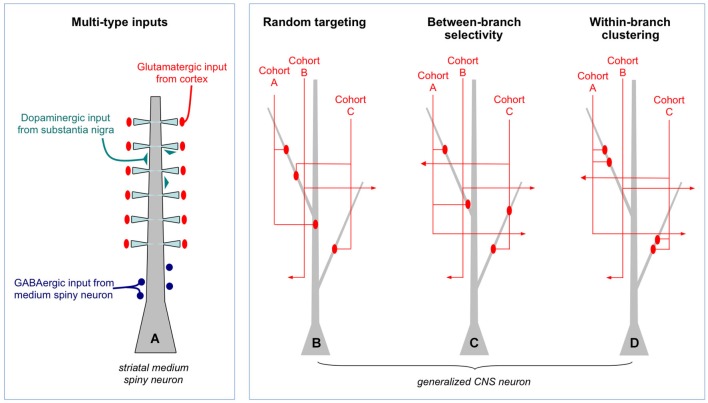
**Random and structured patterns of local connectivity. (A)** Simplified schematic of the inputs to a medium spiny neuron in the striatum. There is a clear structure to the targeting of different types of inputs. Such multi-type structure is reviewed extensively elsewhere and will not be discussed further. **(B)** Generalized CNS neuron (e.g., cortical pyramidal cell) in which inputs of one type only are shown. Within that type, different input cohorts are defined solely on the basis of their activation histories: each cohort (**A** or **B**, among many) has correlated activity whereas different cohorts are uncorrelated. In this example, synapses from three input cohorts are distributed randomly throughout the dendritic field. This is a Braitenberg network. **(C)** Example of between-branch selectivity. Three cohorts have equal access to the leftmost dendrite but only one actually synapses with it. **(D)** Example of within-branch structure. Synapses from each cohort are spatially clustered.

The alternative view is structured connectivity as described in Figure 1. It is framed from the perspective of a postsynaptic cell choosing among potential input partners. A crucial clarification is made in Figure 1A which shows as an example a medium spiny neuron in the striatum. Both the number and dendritic location of synaptic inputs are structured (Smith and Bolam, 1990): glutamatergic input coming from cortex profusely target dendritic spine heads, dopaminergic input coming from substantia nigra sparsely target dendritic shafts and spine shafts, and GABAergic input from other medium spiny neurons target the perisomatic region. Intuitively, the cell would not operate properly if these inputs were randomly scattered on the dendritic field. Yet from the perspective of partner selection, given that the information content and neurochemical identity of the three types of inputs are fundamentally different, the axons did not have equal access to the dendrite during synaptogenesis; molecular markers intrinsic to each input type would have biased them to different postsynaptic compartments. Thus, this particular form of microscale structure, rife throughout the brain (recent example: Petreanu et al., 2009), does not directly address the issue of Peters’ rule and the Braitenberg accident.

What about the patterning within a single type of input (Figures 1B,C,D)? These synapses are by definition carrying the same qualitative information, for example, the location of a visual stimulus on the retina, release the same neurotransmitter(s) and target the same postsynaptic domain, in this case, dendrites. Within each type of input, however, are cohorts that differ in their fine temporal structure: one most active when the visual stimulus is located at 0°, another at 1°, another at 2° etc… If the cohorts obeyed Peters’ rule they would converge or diverge from individual dendrites based solely on geometry, not the degree of co-activation (Figure 1B). Moreover, targeting within a dendritic branch would also be spatially random. Indeed, this arrangement may describe input structure at early developmental stages, immediately following the first wave of synaptic proliferation.

**Figure 2:**
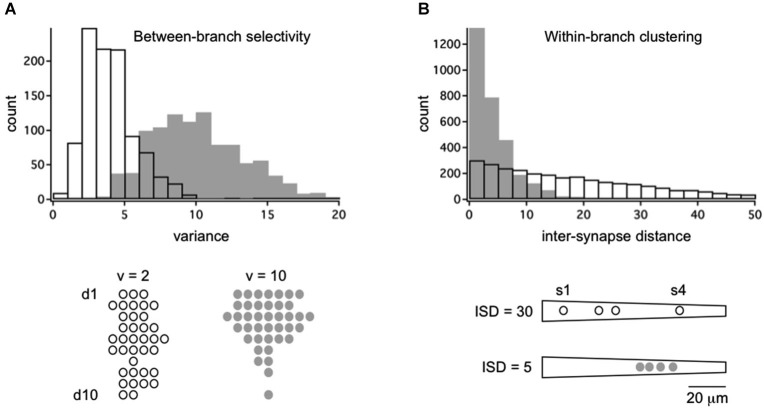
**Framework for the quantitative analysis of between-branch selectivity and within-branch clustering**. **(A)** Distribution of 40 synapses across a population of 10 dendrites with random (open bars/symbols) or selective targeting (gray bars/symbols). Synapses were assigned to dendrites by independently sampling from evenly distributed noise (random) or a Gaussian distribution with SD = 4 (selective). Examples of outcomes are shown below for populations with dendrite-to-dendrite variance = 2 or 10. Symbols represent synapses for each of 10 dendrites, d1–d10. **(B)** Distribution of 4 synapses across a single dendrite with random (open bars/symbols) or selective targeting (gray bars/symbols) of dendritic address. Synapses were assigned to an address by independently sampling from evenly distributed noise (random) or a Gaussian distribution with SD = 10 (selective). Intersynapse distance (ISD) was measured along the dendritic path, yielding three ISDs per dendrite (total of 3000 ISDs). Example of outcomes are shown below for dendrites with mean ISD = 30 or 5. Symbols represent dendritic address.

If random structure does occur it could be a tabula rasa for experience-dependent refinement (Kalisman et al., 2005). At least two kinds of structure are possible. In between-branch selectivity (Figure 1C) independent cohorts innervate or ignore dendritic branches at a higher rate than predicted from geometry. How exactly one defines the local volume is important. An empirically supported definition is to consider the bulk spatial density of axons within one spine’s reach of the dendrite (Stepanyants and Chklovskii, 2005). Based on numerous observations that the branch structure of living dendrites is static while their filapodial extensions (precursors to spines) can be motile, this volume is considered the active realm of anatomical remodeling in normal post-developmental circuits (Chklovskii et al., 2004). The results of a simulation based on this rule are shown in Figure 2A. An input cohort of 40 synapses was given equal access to 10 dendrites and asked to choose partners based on a random sample of evenly distributed noise. The simulation was run 1000 times, and the dendrite-to-dendrite variance in number of synapses made was calculated for each run. The resulting distribution of values is narrow with a peak at 2–3 (units of variance = (# synapses per branches)^2^), reflecting the expected outcome that most dendrites received about four synapses each (clear symbols in Figure 2A). In contrast, if partner choice was biased by sampling from a Gaussian distribution that favored certain dendrites over others, the distribution was shifted towards a mean of ~11, reflecting the outcome that certain dendrites were overpopulated by the cohort and others actively ignored (gray symbols in Figure 2A).

Anatomical tests of between-branch selectivity have been an implicit goal of earlier studies (reviewed in White, 2007), and an explicit goal of current ones (see Section Empirical Tests of Input Clustering). In addition, electrophysiological studies have provided relevant data. If cohorts actively chose from equivalent dendrites based on activation history, the connection probabilities between pairs of neurons should be non-random. In studies using paired recordings and laser photostimulation, the connection probability of neighboring visual cortical neurons in layer 2/3 varied with their propensity to receive common excitatory input from layer 4 but was independent of common inhibitory inputs (Yoshimura et al., 2005). In studies using multiple simultaneous whole-cell recordings, connection maps of neuronal triplets in visual cortex were highly non-random with certain motifs over-represented compared to chance (Song et al., 2005). Both studies support the idea that functional cohorts sort over time to the same neuron. It is a small extrapolation to postulate sorting to the same dendrite, where postsynaptic potentials (PSPs) sum locally resulting in output to the soma. An important finding is that the relative weight of each dendrite’s output is not always fixed but can be adjusted by patterned stimulation or enriched experience, a phenomenon termed branch strength potentiation (Losonczy et al., 2008; Makara et al., 2009; Müller et al., 2012). This is but one example of how branch selectivity might contribute to the encoding of learning and memory (Legenstein and Maass, 2011).

The other potential structure is within-branch clustering (Figure 1D). For the cognate simulation shown in Figure 2B, four synapses from the same cohort were given access to a 100 micron long dendrite. With random sampling from an even distribution (open symbol), the distribution of inter-synapse distances (ISDs) was broad and relatively flat, with nearly as many dendrites harboring synapses separated by ~20 or more microns as there were dendrites harboring spatially clustered synapses. In contrast, if dendritic address was biased by sampling from a Gaussian distribution that favored certain locations over others, the ISD distribution was heavily compressed with a mode of <3 microns and no dendrites at all harboring dispersed synapses (gray symbols). For the remainder of this review we focus on these hypothetical dendritic input clusters, their functional implications, and recent anatomical tests of their existence.

## Input clustering hypothesis

There are two general models for how neurons integrate synaptic input. In a global integration model, PSPs resulting from individual synapses sum linearly at the soma. Supporting this, studies combining whole-cell patch clamp and activation of two dendritic locations using synaptic stimulation or glutamate iontophoresis observed linear summation (e.g., Cash and Yuste, 1999; Araya et al., 2006). If this summation rule holds for physiologically relevant inputs (more than 2), highly structured dendritic addressing as described above should not be essential for neuronal computation. The processing power of neural circuits would come from the intrinsic capacity of linear neurons to perform complex operations such as principle component analysis (e.g., Oja, 1982), compartmentalized electrical and biochemical signaling (at spines), input-specific learning rules, and a massively distributed network (Yuste, 2011).

The alternative is a two-stage integration model in which each dendrite acts as an independent computational subunit capable of supralinear summation. If this is the main operating mode, both branch selection and within-branch clusters should be essential for tapping the full power and storage capacity of circuits. This idea was explored by computational modeling using biophysically and anatomically realistic reconstructions of CNS pyramidal cells (Mel, 1992, 1993; Poirazi and Mel, 2001; Poirazi et al., 2003a,b). For within-branch interactions, co-activation of synapses located within ~40 microns of one another produced a much stronger dendritic response than calculated from the sum of individual activations, whereas those located >60 microns apart summed linearly. This location-dependent supralinearity was a consequence of active conductances, voltage-gated sodium and calcium channels, and NMDA receptors, located within the dendritic membrane. Supralinear summation occurred only in specific input regimes i.e., the parameter space of strength, timing, number and position of synaptic inputs. Indeed, the model predicted linear or weakly sublinear interactions when only two small inputs were activated, in agreement with the results cited above (Araya et al., 2006).

Electrophysiological studies focusing on input regimes predicted to produce supralinearity have found corroborating evidence. In the dendrites of layer 5 pyramidal cells (Polsky et al., 2004), activation of inputs spaced 20–40 microns apart produced an approximately two-fold supralinearity, though only for intermediate activation strengths; the effect disappeared for very weak or very strong inputs. Also consistent with model predictions, blockade of NMDA receptors with APV linearized the response. In recordings from hippocampal CA1 pyramidal cells (Gasparini and Magee, 2006) or striatal medium spiny (Carter et al., 2007) dendrites actively switched between different integration modes (linear vs. supralinear) depending on the input regime. Where dendritic nonlinearities are evident, they are mediated by NMDA spikes/plateau potentials that represent a first level of integration localized to single dendritic branches (Major et al., 2008; Larkum et al., 2009; Polsky et al., 2009; Gómez González et al., 2011; Behabadi et al., 2012; Harnett et al., 2012). Collectively, these data support a two-stage integration model, and highlight a potential role for input clusters as sites of integration and information storage (reviewed in Govindarajan et al., 2006; DeBello, 2008; Larkum and Nevian, 2008; Branco and Häusser, 2010; Magee, 2011; Winnubust and Lohmann, 2012).

## Empirical test of input clustering

In 2001 Poirazi and Mel posed an acid test. They envisioned a postsynaptic neuron integrating four inputs whose synapses were initially randomly scattered on the dendritic field i.e., a Braitenberg network. The experiment consisted of repeated activation of input cohorts A and D, and separately of cohorts B and C. The prediction of their model is that, over time, synapses from paired cohorts will come to reside in input clusters that segregate from the other pair, both within and between branches.

**Figure 3:**
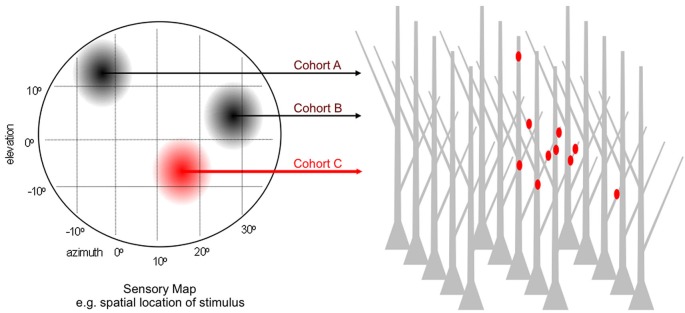
**Paradigm for testing the microanatomical predictions of input clustering**. Generic sensory map representing a 2D feature space e.g., the elevation and azimuth of auditory or visual stimulus. For normally patterned experience, afferents encoding discrete locations within this feature space are co-active over the lifetime of the animal and largely desynchronized with those encoding other locations. Each location corresponds to a cohort, and the entire collection of cohorts goes on to synapse within a dense field of target dendrites. The dendritic address of each synapse is directly visualized and referenced to the activity history, by simultaneous recording sensory-driven responses or by retrospective assignment on the basis of *in vivo* labeling as highlighted for cohort C.

Since 2008 at least nine independent groups have conducted related tests of this general model. These studies used different sensory, motor and memory circuits, different tools for visualization and distinct platforms for quantitative analysis. Most followed a variant of the experimental logic described in Figure 3. In lieu of chronic stimulation the experiments monitored activation history to identify cohorts. For a sensory circuit encoding a particular stimulus feature—e.g., spatial location of auditory, visual or tactile stimulus; orientation of visual bar; frequency of sound—the collection of parallel afferents represents a homogenous input type. Within it, afferents encoding similar values of the feature have a history of high co-activity and those encoding dissimilar values, a history of weak co-activity. Analogous correlations might occur in bursts of spontaneous activity. Thus, the challenge is to record the feature selectivity (or spontaneous activity) of each cohort while simultaneously mapping its dendritic input locations.

### Evidence in support

The first study to use this paradigm was performed in the midbrain of prism-adapted barn owls (McBride et al., 2008). The owls’ auditory space map is computed by integration of binaural inputs that continue change well into juvenile development, which contrasts with visuotopic or somatotopic maps whose inputs are normally fixed early in development. When owls are reared wearing prism glasses that shift the frontal visual field by 19°, a new circuit sprouts within the external nucleus of the inferior colliculus (ICX) and drives adaptive localization behavior. Yet the normal circuit does not wither anatomically but is preserved alongside the learned circuit (Knudsen, 2002), providing an internal control for clustering analysis. Following *in vivo* electrophysiological measurement of auditory-visual spatial receptive fields, co-active axons were labeled with fluorescent tracer, fixed, and imaged with a confocal microscope operating at the diffraction limit. Axodendritic contacts (putative synapses) were identified by requiring volume overlap demonstrated for objects (two-color beads) in physical contact, which excluded the majority of observed touches (Rodriguez-Contreras et al., 2005). Dendritic locations were mapped across hundreds of dendrites and thousands of contacts. Within each dendrite, the inter-contact distance (ICD) to nearest neighbor was measured. ICD distributions from the functionally suppressed zones in prism-adapted or normal juvenile owls were intermediate between those depicted in Figure 2B: most ICDs were <10 microns, however, a significant fraction were >20 microns. In comparison, not a single ICD >20 microns was observed in the functionally strong learned circuit: all of its inputs resided in clusters.

One difference between these results and the *in vitro* findings in mammalian slice experiments is the cluster size was smaller, ~10–20 microns as opposed to ~20–40. Because not all axodendritic contacts identified by LM are synapses, the presence of false positives intercalated between true synapses would bias towards an underestimate of cluster size. Still, bootstrap analysis showed that statistical differences between normal and learned clusters were robust even for high false positive rates that exceeded the error estimates based on co-localization of Homer1, a postsynaptic marker. In total, these results are consistent with the input clustering hypothesis and demonstrate that behaviorally relevant experience can selectively eliminate “lone” synapses from their co-active cohort. Both physical elimination of lone synapses and formation of new co-active synapses within the dendritic neighborhood appeared to be required to account for the observed input distributions.

Other tests of input clustering have identified synapses using cellular-level functional imaging. Kleindienst et al. used *in vivo* two-photon microscopy to visualize spontaneous (synaptic) dendritic calcium transients in cultured hippocampal pyramidal cells from newborn rat pups (Kleindienst et al., 2011). Transients arising from neighboring locations on a dendrite were more often co-active than those arising from distant locations. This propensity for co-activation was quite strong for inter-synapse distances (ISDs) of 16 microns or less, and exhibited a Poisson-like distribution similar to that observed in the functionally weak zones in the owl auditory space map (McBride et al., 2008). Finally, when TTX was applied to the cultures to block activity, the distribution flattened considerably and appeared similar to that shown in Figure 2B (open circles), which suggests that activity is required to maintain non-random (clustered) structure.

Takahashi et al. imaged spontaneous spine calcium transients in cultured CA3 pyramidal cells from 7 day old rat pups and found that the probability of observing spines coactivated within 100 ms was high for neighboring spines but dropped to chance at inter-spine distances greater than 10 microns (Takahashi et al., 2012). Imaging of layer 2/3 pyramidal cells in the barrel cortex of young adult mice confirmed these observations, documented a cluster (hot zone) size of ~8 microns, and demonstrated that clustered spine heads were larger on average than dispersed ones, a proxy indicator of synaptic strength. In a related experiment using adult mice, GluR1 was observed to preferentially insert into neighboring spines following spatial exploration. These results support the idea that correlated activity, over time, leads to the formation and stabilization of clustered inputs.

Makino and Malinow monitored the movement of fluorescently tagged AMPA receptors into spines on the basal dendrites of layer 2/3 pyramidal cells in acute brain slices prepared from juvenile rats (27–36 days old). The potentiation of neighboring spines as indicated by GluR1 insertion was observed to be significantly correlated in 28/95 dendrites after 2 days of normal sensory experience, but only 5/68 dendrites in whisker-trimmed animals (Makino and Malinow, 2011). In contrast, global upscaling as indicated by GluR2 insertion exhibited little or no dendritic compartmentalization in both intact and trimmed animals. These results are consistent with input clustering and also support the notion that global activity reduction/blockade revert the network to an “equal access” situation exhibiting a more random structure (Figure 1B).

Fu et al. used two-photon microscopy to monitor layer 5 pyramidal cells in mouse motor cortex (Fu et al., 2012). As juvenile mice (1 month old) practiced a novel forelimb task over 4 days of training, one third of new spines appeared in clusters (adjacent neighbors), and these were more resistant to elimination than non-clustered new spines, even long after the end of training. When mice were cross-trained as adults on a different task, new clusters emerged and largely segregated from those associated with the first task. These are important findings, consistent with the hypothesis and novel in that they extend both outside sensory systems and to learning in the adult brain. One difference is that the observed cluster size, ~2 microns, is smaller than observed in previous studies.

The studies above focused on excitatory synapses. Chen et al. developed techniques to visualize inhibitory synapses (Chen et al., 2012). Using *in vivo* two-photon imaging of layer 2/3 pyramidal cells in mouse visual cortex and a genetically encoded gephyrin-conjugated fluorophore, inhibitory synapses made on dendritic spines were observed to be considerably more dynamic than those made onto dendritic shafts, both during normal experience and after 2 days of monocular deprivation. Remarkably, a large fraction of dynamic inhibitory synapses were located within 10 microns of other dynamic spine events—ones likely involving excitatory synapses. This observation supports a cluster size of ~10 microns, and suggests that experience-dependent formation and elimination of inhibitory and excitatory synapses may be co-regulated.

Two recent studies employed higher-throughput approaches. In one, Rah et al. used AT to analyze the spatial distribution of thalamocortical synapses onto layer 5 pyramidal cells in mouse somatosensory cortex (Rah et al., 2013). Both between-branch selectivity and within-branch clustering (5–15 microns) were more prevalent than predicted from a random distribution. Because the population of thalamocortical afferents was presumably heterogenous with regards to coactivation history, labeling of functional cohorts within this population will be required to test the most significant predictions of the input clustering hypothesis.

The latest report provides some of the best quantitative analysis to date of cell type selectivity, branch selectivity and input clustering. Druckmann et al. used mGRASP, an optimization of GRASP for mammalian synapses (Kim et al., 2011), to visualize input patterns between presynaptic CA3 and postsynaptic CA1 pyramidal neurons (Druckmann et al., 2014). At the level of individual neurons, the number of synapses per neuron was highly variable and not explained by differences in total surface area of the dendritic field. Across dendritic branches of a given neuron, the number of synapses per branch was highly variable and deviated from a purely random distribution in 22 of 28 neurons analyzed. This was not due to branch-to-branch variations in overall synapse density, which were found on the basis of spine counts to be similar, as expected from previous work. The authors then applied a linear form of Peters rule to investigate whether local variation in axonal density could predict branch-to-branch variation in synapse density and found little evidence in support, except for a small number of branches with very low synapse and axonal density. In total, these data provide a direct demonstration of structured branch selectively consistent with the illustrations in Figures 1C, 2A.

The authors went on to analyze within-branch clustering and found significant deviations from a random distribution in 17 of 27 neurons. All exhibited an overabundance of short ISDs consistent with the illustrations in Figures 1D, 2B. One important caveat is that the axonal inputs that gave rise to these clusters could not be reliably traced to their source(s) and therefore likely involved a mix of input sources whose temporal correlations are unknown.

Finally, the authors repeated the experiments by labeling only “temporally matched neurons”, defined as arising during the same developmental window. For these sparsely labeled datasets, input clustering was significantly enhanced over that expected by chance. In total, these results are consistent with the input clustering hypothesis and also illustrate the importance of a rigorous quantitative framework, and large *n* numbers, to distinguish between structured and random connectivity. Because the relationship between the actual co-activity histories and/or feature selectivities among temporally matched neurons in the hippocampal circuit is not well-understood, and not likely as straightforward as topographic organization in sensory systems (Figure 3), these results do not establish proof of the input clustering hypothesis.

### Evidence not in support

Not all studies have found evidence of clustering. Three studies by Konnerth et al. used *in vivo* two-photon imaging of dendritic calcium transients evoked by sensory stimulation. No clustering was apparent on layer 2/3 cells in mouse visual cortex (Jia et al., 2010) or layer 2 neurons of mouse vibrissal cortex (Varga et al., 2011), and related experiments employing an imaging method with single spine resolution found no evidence of clustering in layer 2/3 cells in mouse auditory cortex (Chen et al., 2011). In all three studies there were clear examples in which calcium signals evoked by similar orientations of visual stimuli, individual whiskers, or frequencies of sound (respectively) were located on different dendrites, and others in which signals evoked by different feature values occurred on the same dendrite, often in close proximity. That activity originating at one location along the sensory epithelium would provide synaptic input to more than one dendrite of a higher order neuron is consistent with both input clustering and global integration models. However, the observation of freely intermingled synapses encoding all aspects of feature space is only consistent with a global integration model in which neurons acquire receptive fields by integrating spatially distributed synaptic inputs.

A similar result was observed in area 17 (V1) of cat visual cortex by da Costa and Martin. The authors used correlated light and electron microscopy to analyze the distribution of thalamic synapses onto layer 4 spiny stellate cells (da Costa and Martin, 2011). Thalamic axons were labeled by focal injections of biotinylated dextran amine tracer at a matched location within the visuotopic space map, an experimental design very similar to that employed for the owl studies. 191 contacts made onto four spiny stellate cells were identified by LM “whenever a gap between a labeled dLGN axon and a labeled dendrite could not be discerned”. Retrospective EM on 50 of these contacts revealed that only 14 were actual synapses, a high false positive rate roughly consisted with other studies. Fully half of the unambiguous false positives (15/30) were found not to be in contact at the EM level. This is an important finding because it suggests that application of the overlap volume criteria described in Rodriguez-Contreras et al. (2005) and used to study input clustering in the owl (McBride et al., 2008) has utility in reducing—not eliminating—false positives from the dataset.

Da Costa and Martin went on to demonstrate that neither the LM-identified contacts or EM-identified synapses clustered on dendrites. Bootstrap analysis confirmed this observation. They conclude that the receptive fields of these layer 4 spiny stellate are determined by the synchronous firing of a relatively small number (188/cell) of thalamic inputs that are distributed randomly throughput the dendritic field.

## Conclusions

**Figure 4:**
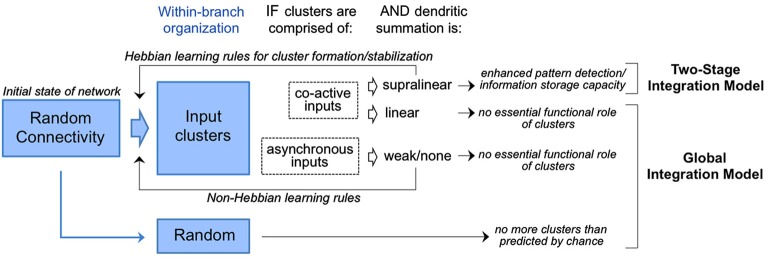
**Competing models for the interpretation of input clusters**. Random connectivity as the initial state of the network is consistent with both models. In circuits where input clusters are observed with prevalence higher than chance (Figure 2), the functional implications depend on the activity histories and mode of dendritic integration. If clusters are of co-active inputs and found to drive supralinear summation, they are predicted to enhanced storage capacity and pattern detection in accordance with the two-stage integration model. In this model, cluster formation/stabilization would rely on Hebbian mechanisms as reviewed in Winnubust and Lohmann (2012). In contrast, clusters are of co-active inputs but found to drive linear summation, or of asynchronous inputs operating outside the dendrites’s temporal integration window, then no essential functional role is predicted in accordance with the global integration model. Formation/stabilization of clusters in this model would rely on non-Hebbian mechanisms.

The first wave of anatomical tests of the experiment proposed in 2001 by Poirazi and Mel have materialized with most, not all, groups finding evidence in support. This issue is hardly resolved. The rules for dendritic integration in many of the cell types used these experiments are not well understood, thus, the more general question of global vs. two-stage integration models remains unclear. Various interpretations of the functional significance of input clusters within this context are summarized in Figure 4. It is of course possible that clustering is used in certain cell types and not others. One could speculate that the adaptive value (evolutionarily) of clustering might be greater for neurons tasked with integrating complex and fluid information streams (e.g., the owl auditory space map or the mammalian hippocampal-neocortical system) than for neurons providing more of a throughput role (e.g., early sensory pathways). Even if true, this speculation does not fully account for the divergence of empirical results.

Where clustering has been observed, one consistent finding is a dendritic window of ~10 microns, somewhat smaller than the window observed for supralinear interactions *in vitro* using multispot uncaging or photactivation. This leaves open a crack in the interpretation of clusters as sites of supralinear integration. The ~10 micron window does match well with the spatial range of intracellular biochemical signaling pathways that lower the threshold for LTP (long-term potentiation) among neighboring synapses (Harvey and Svoboda, 2007; Harvey et al., 2008). Thus, a non-exclusive interpretation for the functional role of input clusters is to promote coordinated regulation of synaptic plasticity among co-active inputs (Figure 4).

In light of these results new challenges arise. First, more data is needed. In some cases the quantitative analysis of between-branch selectivity and within-branch clustering has been limited by relative paucity of primary data owing to the intense labor involved in data collection. The simulations shown in Figure 2 provide a framework for analysis, but also caution that discerning random vs. non-random connectivity will likely require complete reconstructions of 10–100s of neurons, 100–1000s of dendrites, and perhaps millions of synapses. This is a tall order for microscale connectomics, though one within reason. For example, a separate prediction of the two-stage integration model was successfully addressed via serial section EM (Katz et al., 2009). One advantage of the emerging high-throughput connectomics methods over standard confocal or two-photon imaging is the high reliability in identifying all synapses within the circuit. This trade-off between throughput capacity and reliability is a factor to weigh in experimental design. Very high-throughput EM imaging is possible (Hayworth, 2012) though not needed for this particular question, and is for now limited by the annotation bottleneck. An out-of-box strategy to sequence the connectome could fast track brain-wide mapping of all neuron-neuron connections (Zador et al., 2012), applicable to many important questions though perhaps not those of dendritic addressing.

Another challenge is to link structure and function, far more difficult task for the brain than other organs (Lichtman and Denk, 2011). For example, even a static synaptic network encodes multiple functional circuits due to state changes mediated by neuromodulators operating on a paracrine scale (Bargmann and Marder, 2013). Yet the need to integrate structural and functional connectomics data is also a ripe opportunity. Prospective recordings of neuronal activity and durable tagging of functionally defined circuit elements, including projection axons, will greatly enhance understanding of the computations performed by connection motifs found in the wiring diagram. New tools for precisely manipulating the activity of circuit elements *in vivo* should provide rigorous tests of these functional motifs. Indeed, the acid test of input clustering as originally proposed has been infeasible for lack of such tools. Thus there is clear need for multiple technologies brought to bear on the same set of problems, for creative digital synthesis of those layers of data, and ultimately, for large-scale simulation.

Another challenge will be to invest resources in a diverse collection of neural circuits across brain regions and species. Genetically accessible models such as the worm, fly and mouse can be approached with the broadest range of tools. In addition, systems neuroscience has amassed very good understanding of computations in a number of other systems that are nonetheless fully approachable with many of the new methods. If the goal is to link structure and function it makes sense to put effort into brain circuits whose computations are both sophisticated and known. For example, hypotheses for connectome-based learning mechanisms have been proposed for the songbird (Seung, 2009) and barn owl (DeBello and Knudsen, 2001), two widely used behaviorally relevant models for information processing and plasticity. Success with these circuits could cross-pollinate with parallel efforts in mammalian cortex.

The payoff is the potential to uncover common mechanisms of learning in healthy brain circuits. Such knowledge will likely be essential to understand and treat dysfunctions arising from disease or trauma. Indeed, many neurodevelopmental or degenerative syndromes including autism and schizophrenia are suspected to result from pathologies occurring at the level of microscale wiring. Yet our knowledge of this level of brain structure is primitive.

Finally, large-scale efforts are underway to promote advances in neuromorphic computing, including the DARPA SyNAPSE program, the Human Brain Project, the Blue Brain Project and Spaun (Eliasmith and Trujillo, 2014). Integrated structural and functional connectome data would appear to hold transformative potential for these endeavors.

## Conflict of interest statement

The authors declare that the research was conducted in the absence of any commercial or financial relationships that could be construed as a potential conflict of interest.
